# Pilot Testing the Feasibility of a Game Intervention Aimed at Improving Help Seeking and Coping Among Sexual and Gender Minority Youth: Protocol for a Randomized Controlled Trial

**DOI:** 10.2196/12164

**Published:** 2019-02-15

**Authors:** Robert WS Coulter, Jordan M Sang, William Louth-Marquez, Emmett R Henderson, Dorothy Espelage, Simon C Hunter, Matthew DeLucas, Kaleab Z Abebe, Elizabeth Miller, Brooke A Morrill, Kimberly Hieftje, Mark S Friedman, James E Egan

**Affiliations:** 1 University of Pittsburgh Pittsburgh, PA United States; 2 College of Health and Human Performance University of Florida Gainesville, FL United States; 3 School of Psychological Sciences and Health University of Strathclyde Glasgow United Kingdom; 4 Schell Games Pittsburgh, PA United States; 5 Yale University New Haven, CT United States

**Keywords:** sexual and gender minorities, adolescent, video games, feasibility studies, help-seeking behavior, adaptation, psychological, alcohol drinking, cigarette smoking, vaping, mental health, randomized controlled trial

## Abstract

**Background:**

Sexual and gender minority youth (SGMY; eg, lesbian, gay, bisexual, and transgender youth) experience myriad substance use and mental health disparities compared with their cisgender (nontransgender) heterosexual peers. Despite much research showing these disparities are driven by experiences of bullying and cyberbullying victimization, few interventions have aimed to improve the health of bullied SGMY. One possible way to improve the health of bullied SGMY is via a Web-accessible game intervention. Nevertheless, little research has examined the feasibility of using a Web-accessible game intervention with SGMY.

**Objective:**

This study aimed to describe the protocol for a randomized controlled trial (RCT) pilot, testing the feasibility and limited efficacy of a game-based intervention for increasing help-seeking–related knowledge, intentions, self-efficacy, behaviors, productive coping skills use, and coping flexibility and reducing health risk factors and behaviors among SGMY.

**Methods:**

We enrolled 240 SGMY aged 14 to 18 years residing in the United States into a 2-arm prospective RCT. The intervention is a theory-based, community-informed, computer-based, role playing game with 3 primary components: encouraging help-seeking behaviors, encouraging use of productive coping, and raising awareness of Web-based resources. SGMY randomized to both the intervention and control conditions will receive a list of SGMY-inclusive resources, covering a variety of health-related topics. Control condition participants received only the list of resources. Notably, all study procedures are conducted via the internet. We conveniently sampled SGMY using Web-based advertisements. Study assessments occur at enrollment, 1 month after enrollment, and 2 months after enrollment. The primary outcomes of this feasibility study include implementation procedures, game demand, and game acceptability. Secondary outcomes include help-seeking intentions, self-efficacy, and behaviors; productive coping strategies and coping flexibility; and knowledge and use of Web-based resources. Tertiary outcomes include bullying and cyberbullying victimization, loneliness, mental health issues, substance use, and internalized sexual and gender minority stigma.

**Results:**

From April to July 2018, 240 participants were enrolled and randomized. Half of the enrolled participants (n=120) were randomized into the intervention condition and half (n=120) into the control condition. At baseline, 52.1% (125/240) of the participants identified as gay or lesbian, 26.7% (64/240) as bisexual, 24.2% (58/240) as queer, and 11.7% (28/240) as another nonheterosexual identity. Nearly half (113/240) of participants were a gender minority: 36.7% (88/240) were cisgender boys, and 16.3% (39/240) were cisgender girls. There were no differences in demographic characteristics between intervention and control condition participants.

**Conclusions:**

Web-accessible game interventions overcome common impediments of face-to-face interventions and present a unique opportunity to reach SGMY and improve their health. This trial will provide data on feasibility and limited efficacy that can inform future Web-based studies and a larger RCT aimed at improving health equity for SGMY.

**Trial Registration:**

ClinicalTrials.gov NCT03501264; https://clinicaltrials.gov/ct2/show/NCT03501264 (Archived by WebCite at http://www.webcitation.org/72HpafarW)

**International Registered Report Identifier (IRRID):**

DERR1-10.2196/12164

## Introduction

### Background

Sexual minority youth (eg, lesbian, gay, bisexual, or queer youth) and gender minority youth (ie, youth who identify as a gender different from their sex assigned at birth) experience myriad substance use and mental health disparities [1-27]. In particular, sexual orientation–related disparities have been known for over 20 years [1]: sexual minority youth, compared with heterosexual youth, have approximately 176% higher odds of cigarette use, 155% higher alcohol use, 34% higher heavy alcohol use (eg, binge drinking), and 56% higher marijuana use [2]. Currently, many of these sexual orientation–related disparities appear to be growing larger [3,4]. Similarly, compared with cisgender (ie, nontransgender) youth, gender minority youth have significantly higher use of cigarettes, alcohol, and marijuana [5,27]. These sexual and gender minority youth (SGMY) disparities are also present for novel substances, such as electronic cigarette use [5]. Regarding mental health disparities, SGMY have significantly higher anxiety, depression, and suicidality [20-27]. Meta-analyses show that sexual minority youth, compared with heterosexual youth, have 96% higher odds of having suicidal thoughts, 120% higher odds of making suicide plans, and 218% higher odds of making suicide attempts [20]. Altogether, these substantial and persistent health disparities make SGMY a priority population for interventions that attempt to reduce health inequities.

SGMY also experience disparities in bullying and cyberbullying victimization compared with their cisgender heterosexual peers [5,27-33]. For example, according to the 2015 Youth Risk Behavior Survey (YRBS), gay, lesbian, and bisexual adolescents (compared with heterosexuals) had nearly doubled the prevalence of bullying and cyberbullying victimization [33]. Importantly, research shows that these bullying disparities also contribute to SGMY disparities in substance use and mental health issues [5,13,27,34,35]. Therefore, interventions that help bullied SGMY and reduce bullying victimization may, in turn, reduce substance use and mental health disparities.

In addition to this greater prevalence of bullying, SGMY have unique factors that also contribute to health disparities. When SGMY are bullied, in addition to the typical fears of disclosing their bullying victimization experiences to others, they often fear having to disclose their sexual and gender minority (SGM) status to adults, thereby putting them at risk for further discrimination and harassment [36,37], likely preventing them from reaching out for help. Even when SGMY consider suicide, large proportions of them do not seek help [38], and sexual minority youth have more trouble than heterosexuals with identifying people to talk to about their emotional worries [39]. Moreover, compared with heterosexuals, sexual minority youth are more likely to use nonproductive coping strategies (eg, self-blaming, giving up, ignoring problems, and worrying) to manage the stressors in their lives [40,41], which likely exacerbates their health. Thus, interventions that aim to improve help-seeking behaviors and productive coping strategies (eg, solving problems, seeking relaxing diversions, and being physically active) among bullied SGMY may substantially reduce substance use and mental health disparities.

Notably, few interventions have been rigorously tested to examine whether they are efficacious in reducing substance use and mental health disparities among SGMY [42]. Moreover, 1 possible way to improve the health of bullied SGMY is via a Web-accessible (ie, downloadable via the internet) game intervention. Game interventions can be easily accessible through the internet, thereby providing an effective way to reach large numbers of SGMY, including SGMY living in rural areas and high structural stigma locations (ie, areas with less SGM-inclusive policies, institutions, and attitudes). Furthermore, Web-based game interventions are advantageous because some SGMY may be *out* to only a few people offline, making recruitment and attendance in face-to-face interventions for SGMY quite challenging [43,44]. Web-accessible interventions may be accessed by those who are insufficiently supported in offline programs [45-47]. In addition, Web-accessible gaming programs about sex [48,49], mental health [50], alcohol use [51-55], smoking [56-58], and asthma [59-62] are effective for youth in general. The Web-based environment is also relatively safe for LGB youth to gain coping skills [63,64]. Other advantages of Web-accessible interventions are increased fidelity and cost-effectiveness [65-71]. Overall, Web-accessible game interventions overcome common impediments of face-to-face interventions and present a unique opportunity to reach SGMY to improve their health. Little research, with few exceptions [72,73], has examined the feasibility of using a Web-accessible game intervention with SGMY populations. Therefore, pilot testing the feasibility of such a study is critical for successfully conducting a large-scale intervention. Feasibility comprises a wide range of topics [74], such as the testing of implementation procedures—how well the study was implemented as planned; intervention demand—the actual use of intervention among participants; intervention acceptability—how the participants react to the intervention; intervention integration—how well the game fits into the participants’ lives; intervention adaptation and expansion—the changes necessary for future iterations of the intervention and translation into new environments; and finally, limited efficacy testing—can be used to get estimates for variability and precision to power a larger randomized controlled trial (RCT). To adequately address the multiple dimensions of feasibility related to a game-based intervention tailored to SGMY, a carefully planned pilot trial is needed.

### Study Aims

This paper describes the protocol for a pilot RCT assessing the feasibility and limited efficacy of the game-based intervention to increase help-seeking–related knowledge, intentions, self-efficacy and behaviors, productive coping skills use, and coping flexibility and reduce health risk factors and behaviors among SGMY. The primary, secondary, tertiary, and exploratory aims of the study are described below.

#### Primary Aim

Our primary aim is to evaluate implementation procedures for the RCT of the game-based intervention as well as to determine the level of game demand and game acceptability. We hypothesize having high implementation fidelity, game demand, and game acceptability (see Table 1 in the Methods section for specific targets).

#### Secondary Aim

Our secondary aim is to test the limited efficacy for the game in increasing the following short-term outcomes: help-seeking intentions, self-efficacy, and behaviors; productive coping strategies and coping flexibility; and knowledge and use of Web-based resources. We hypothesize that compared with the control participants, the intervention participants would have greater improvements in all short-term outcomes.

#### Tertiary Aim

Our tertiary aim is to test the limited efficacy for the game in reducing long-term outcomes: bullying and cyberbullying victimization; loneliness; mental health issues (ie, stress, anxiety, depression, and suicidality); substance use; and internalized SGM stigma. We hypothesize that intervention participants, compared with control participants, will have greater decreases in all long-term outcomes.

#### Exploratory Aim

We have built in an exploratory aim meant to better our understanding of participants’ responses to the game and research procedures and how to improve both. This exploratory aim concerns implementation procedures, integration, and the adaptation and expansion of the game. Given the exploratory nature of this aim, we have no a priori hypotheses.

## Methods

### Study Design

The purpose of this research study is to pilot a 2-arm RCT of a game-based intervention to improve help-seeking behaviors and productive coping strategies to reduce substance use, victimization, and mental health issues among SGMY. The study is led by a team with expertise in SGMY research at the Center for LGBT Health Research in the University of Pittsburgh’s Graduate School for Public Health, University of Pittsburgh (Principal Investigators: Friedman and Egan). We engage in team science by partnering with an expert in bullying research, help-seeking research, an interventionist, a biostatistician, a health and game researcher, and a professional game development company. The study is funded by the Eunice Kennedy Shriver National Institute of Child Health and Human Development at the National Institutes of Health (R21HD083561) and is registered as a clinical trial (NCT03501264) [75].

### Study Population and Study Flow

**Figure 1:**
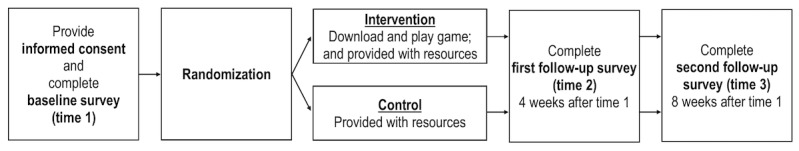
2-Arm randomized controlled trial design and data collection schedule.

All study procedures related to screening, consenting, surveying, and reminding are completed using REDCap, a free and secure Health Insurance Portability and Accountability Act-compliant website for managing Web-based surveys and databases. Study participant flow is depicted in Figure 1.

First, to determine eligibility, respondents completed a brief Web-based self-reported screening questionnaire before entering in this study. Respondents were eligible if they were English literate, lived in the United States of America, were aged between 14 to 18 years, had experienced bullying or cyberbullying victimization in the past year, had a sexual minority identity (ie, gay, lesbian, bisexual, or queer) or a gender minority identity (ie, considered themselves to be transgender or nonbinary), had a personal computer or Mac laptop or desktop computer where they could download games, and had an email address. Eligible respondents were then directed to a Web-based informed consent form. Participants voluntarily consented using a *click-to-consent* procedure. To protect participants from having to reveal their sexual or gender minority identities to their caregivers, thereby potentially putting them in harm’s way, we received a waiver of parental consent [76]. This allowed participants to self-consent. Ineligible respondents were thanked for their time and no additional contact was made.

Once eligible respondents agreed to voluntarily participate in the study, they were emailed a link to the baseline (T1) survey to complete. The T1 survey contained 24 pages with a median of 8.5 items per page (mean 10.3; range 3-26). After completion of the T1 survey, the study team randomized participants to intervention or control conditions (full descriptions of conditions are provided in later sections). Immediately after randomization, participants in the intervention condition automatically received an email with a REDCap survey link containing information that guided participants through the procedures for downloading and installing the game intervention onto their desktop or laptop computers with Microsoft Windows or Apple Mac operating systems. Every 3 days thereafter, participants were automatically sent up to 5 email reminders to download the game. Moreover, 1 day after randomization, participants in both the intervention and control conditions were automatically sent a list of resources related to study outcomes.

The first follow-up (T2) survey was activated 4 weeks after T1 survey completion and remained open for 4 weeks. The T2 survey was similar to the T1 survey; however, participants in the intervention condition who self-reported having played the game also completed questions about their gaming experience. The T2 survey contained 26 pages with a median of 8.5 items per page (mean 11.4; range 3-47).

The final follow-up survey (T3) is activated 8 weeks after T1 survey completion and remains open for 8 weeks. The T3 survey is similar to the T1 survey; however, intervention condition participants who do not complete the gaming experience questions at T2 but self-report having played the game in the T3 survey will be asked questions about their gaming experience at T3. The T3 survey contained 26 pages with a median of 8.5 items per page (mean 11.4; range 3-47).

For each survey, up to 5 email reminders to complete surveys are automatically emailed to participants every 4 days. While completing each survey, participants were able to change their answers by clicking a *back* button. The following incentives are given after the completion of each survey: US $10 for T1; US $25 for T2; and US $50 for T3. At the end of each survey, participants select if they wanted a gift card to Apple iTunes or Google Play.

### Recruitment

Participants were conveniently sampled and recruited throughout the United States using website advertisements posted on social media platforms. This passive approach allowed SGMY from multiple geographic locations (eg, rural and urban, and East and West) to enroll in the study without overextending our limited resources. Facebook was our primary recruitment site. Facebook is an appropriate recruitment platform because it is highly utilized by adolescents; approximately 71% of teens use Facebook [77]. We created a formal side-bar Facebook ad (in-line ads were not used). We also recruited participants from Instagram using the same advertisements. We also recruited participants from SGM-related Web-based gaming groups, such as Geeks OUT, Gay Geeks (Facebook group), GaymerX (Facebook group), Transmission Gaming, and Reddit Gaymer forums. We advertised via Pitt+Me, a community hosted by the University of Pittsburgh comprising patients, volunteers, and researchers working together as partners in research and clinical trials to advance health care. Finally, we advertised on a dedicated Facebook page for the study. Only study team members were able to post comments on this page. An individual who was interested in participating clicked on the advertisement and was directed to the Web-based screening questionnaire. To ensure representation of both sexual and gender minorities, enrollment was monitored weekly. Specific ads were created and used to target underrepresented groups. Depending on the prior week’s enrollment numbers, we tailored which ads were used for the upcoming week.

### Randomization

We used permuted block allocation (using blocks of several sizes) to randomize individual participants to the intervention or control conditions. The permuted blocks were created using the ralloc package for Stata. Randomization was performed in REDCap using the Randomization Module.

### Control Materials

All participants randomized into the control condition received a list of national SGM-inclusive resources. This list included general lesbian, gay, bisexual, transgender, and queer (LGBTQ) resources (eg, GLAAD resource list), LGBTQ bullying resources (eg, The Trevor Project), general bullying victimization resources (eg, StopBullying.gov), child abuse resources (eg, Child Abuse Resource Center), dating violence resources (eg, National Domestic Violence Hotline), suicide and mental health resources (National Suicide Prevention Lifeline), substance use resources (eg, National Institute of Drug Abuse for Teens), LGBTQ homelessness (eg, True Colors Fund), and LGBTQ emergency hotlines (eg, Gay, Lesbian, Bisexual, and Transgender National Help Center Hotline). These materials were delivered via email the day after participants were randomized to the control condition. In addition, after completion of the final T3 survey, control participants were offered a free download of the intervention game. No additional follow-up was initiated to assess their game use, game satisfaction, or changes in outcomes.

### Intervention Materials

Participants assigned to the intervention condition received a list of SGM-inclusive resources (the same as the control condition materials) and the game intervention. After randomization, intervention condition participants received an email with a link and instructions on how to download the game. Participants were asked to download the game to their computer to play it. The game intervention was based on empirical interviews with SGMY about their gaming preferences, undergirded by etiologic and behavioral change theories and built in collaboration with expert educational game developers.

#### Gaming Preferences of Sexual and Gender Minority Youth

**Figure 2:**
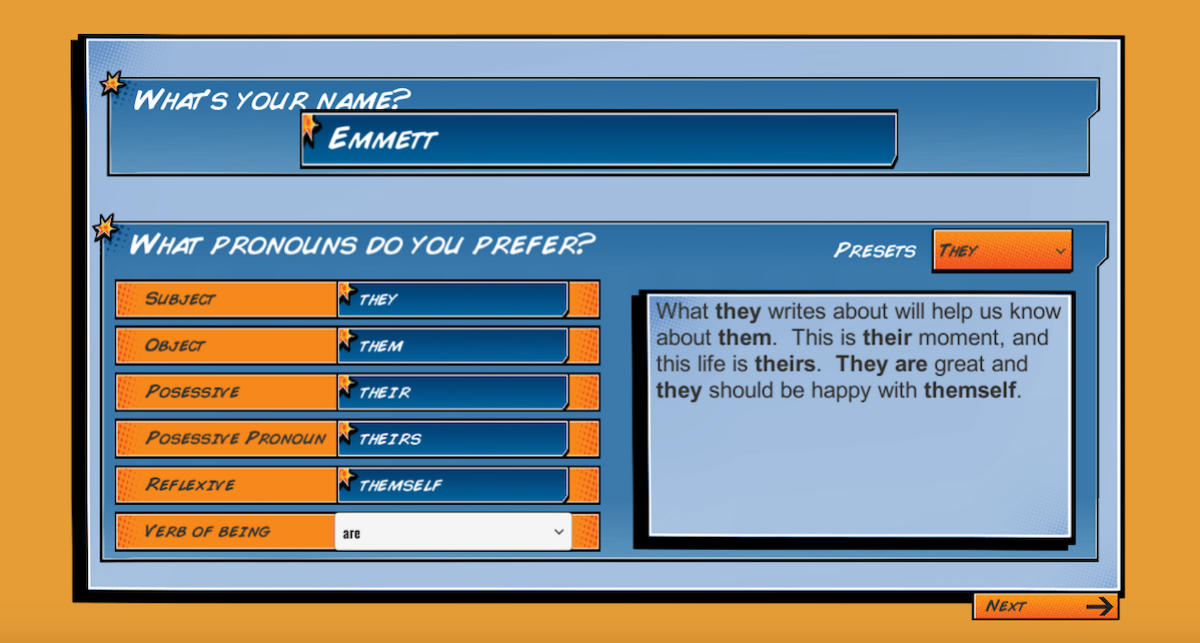
Game intervention name and pronoun selection.

**Figure 3:**
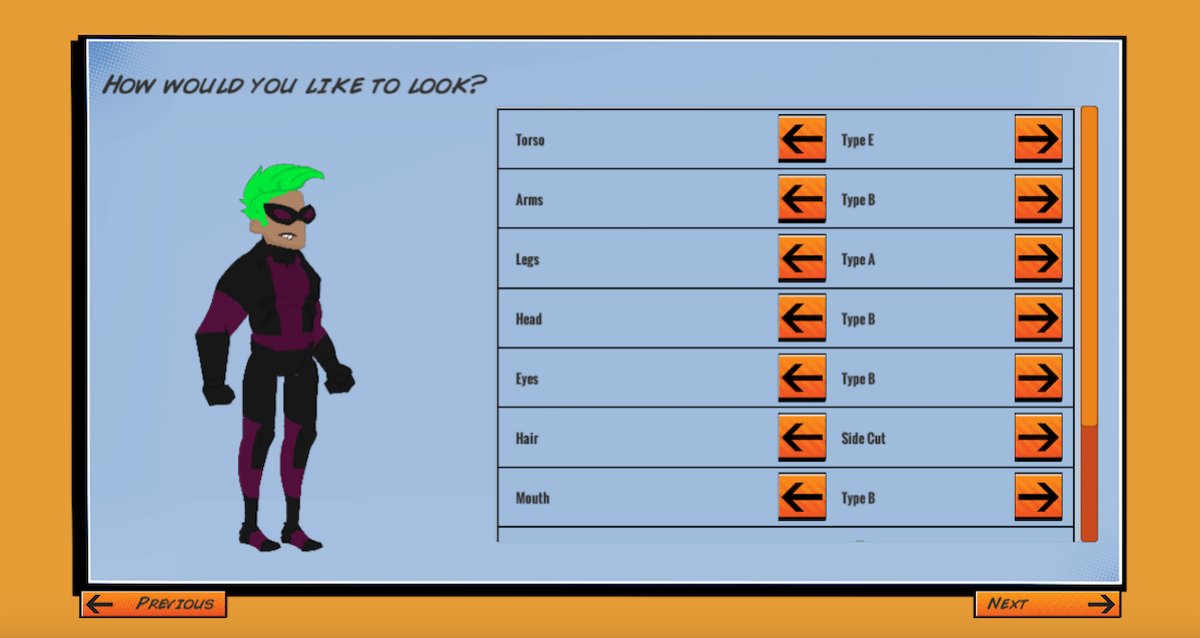
Game intervention character customization.

Before developing the game, we conducted one-on-one, in-depth interviews with 20 SGMY about their gaming preferences (publication of these results is currently underway). Most SGMY enjoyed playing action-oriented games, and when asked what they liked about their favorite games, the most common response concerned the ability to personalize characters. Therefore, in the game, we incorporated selection of pronouns (Figure 2) and character customization, including skin color, hair, body type, and clothing (Figure 3). SGMY liked engaging, unique storylines, as well as challenging (but not too challenging) mini objectives and missions. Having a multiplayer aspect of the game that requires teamwork and interaction with others was also mentioned. We also incorporated these elements into the game.

#### Theoretical Underpinnings

**Figure 4:**
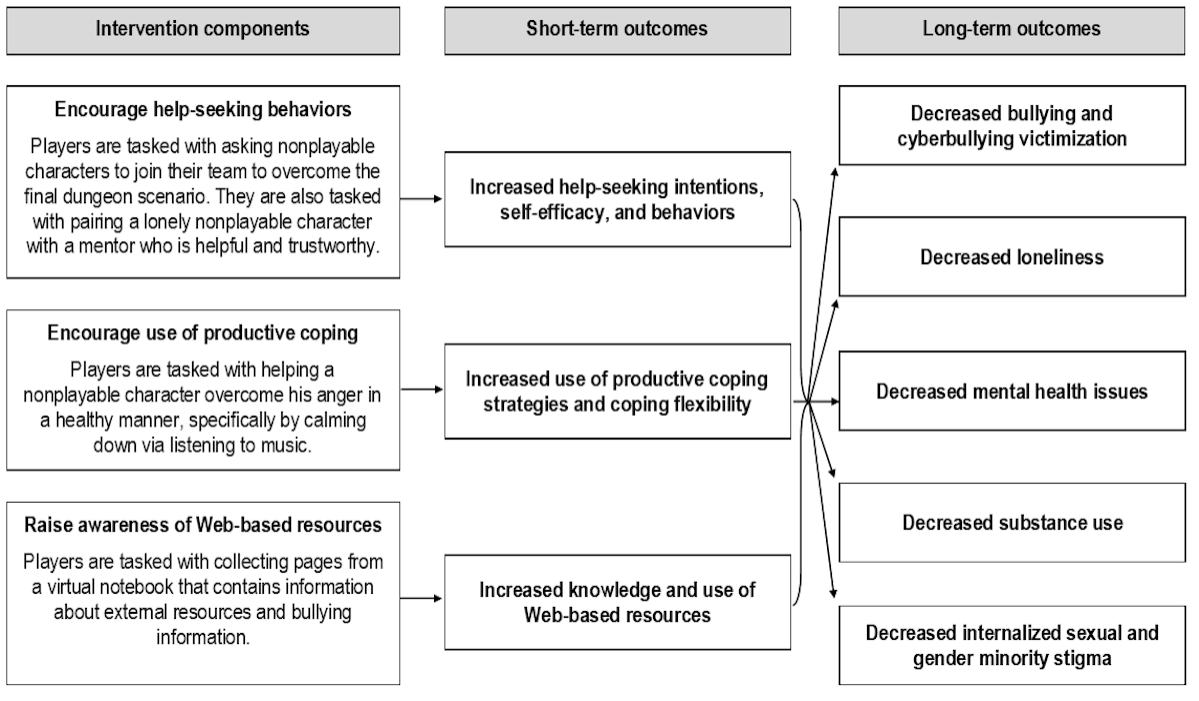
Game intervention conceptual model.

The conceptual model of the game intervention (Figure 4) is based on 3 theories that inform the etiology and behavioral change strategies of our short-term and long-term outcomes. First, social cognitive theory [78,79] suggests that behavior change (eg, help-seeking behaviors) is facilitated by developing self-efficacy and skills. Self-efficacy and social skills can be achieved through behavioral rehearsal, witnessing outcomes of their choices, and feedback [80-82]. Second, stress and coping theory [83] suggests that specific types of appraisals predict different coping strategies. With bullying, youth who blame themselves, perceive little control, and view the situation primarily as a threat as opposed to a challenge are likely to engage in nonproductive coping [84-87]. On the other hand, identification of the problem, direct problem solving, and help seeking constitutes productive coping [88]. Third, the social and emotional learning framework [89] identifies 4 main competencies to promote positive health outcomes: awareness of self and others, responsible decision making, positive attitudes and values, and social interaction skills. We considered each of these theories during the process of developing the core game mechanics of the player experience.

**Figure 5:**
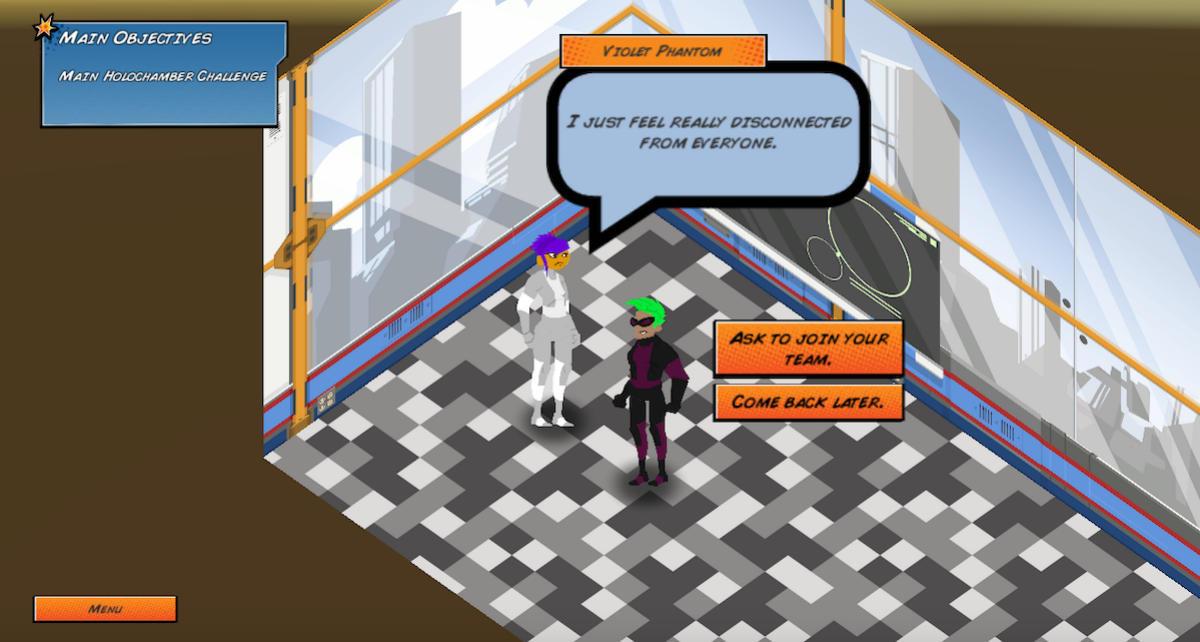
Interaction with a nonplayable character who is lonely.

**Figure 6:**
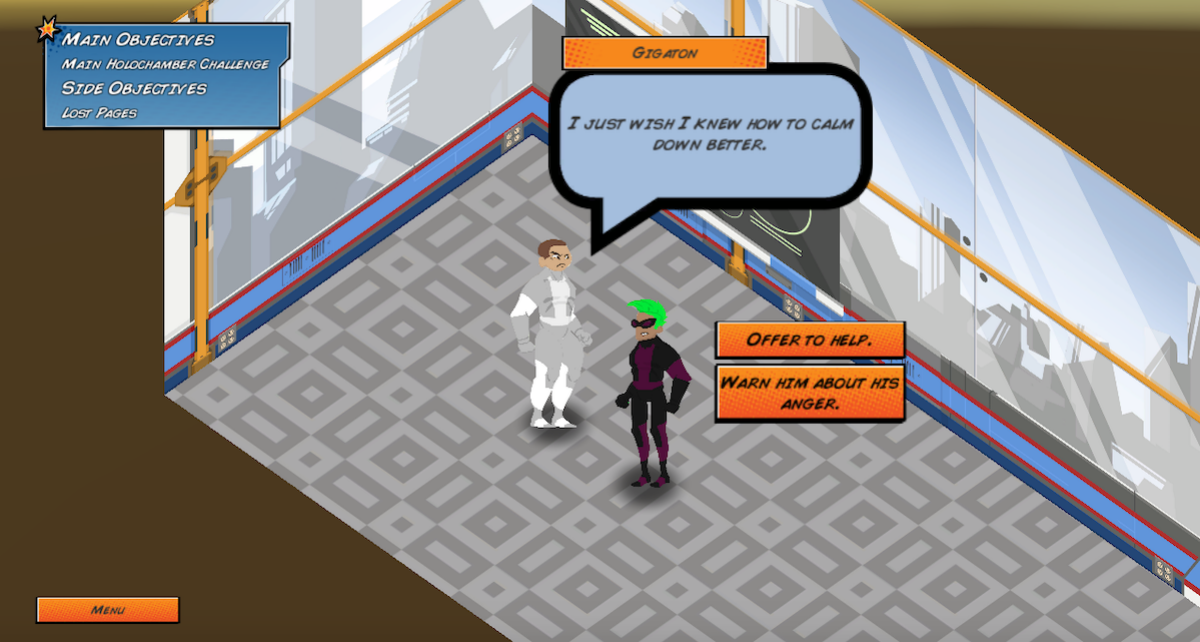
Interaction with a nonplayable character who is angry.

**Figure 7:**
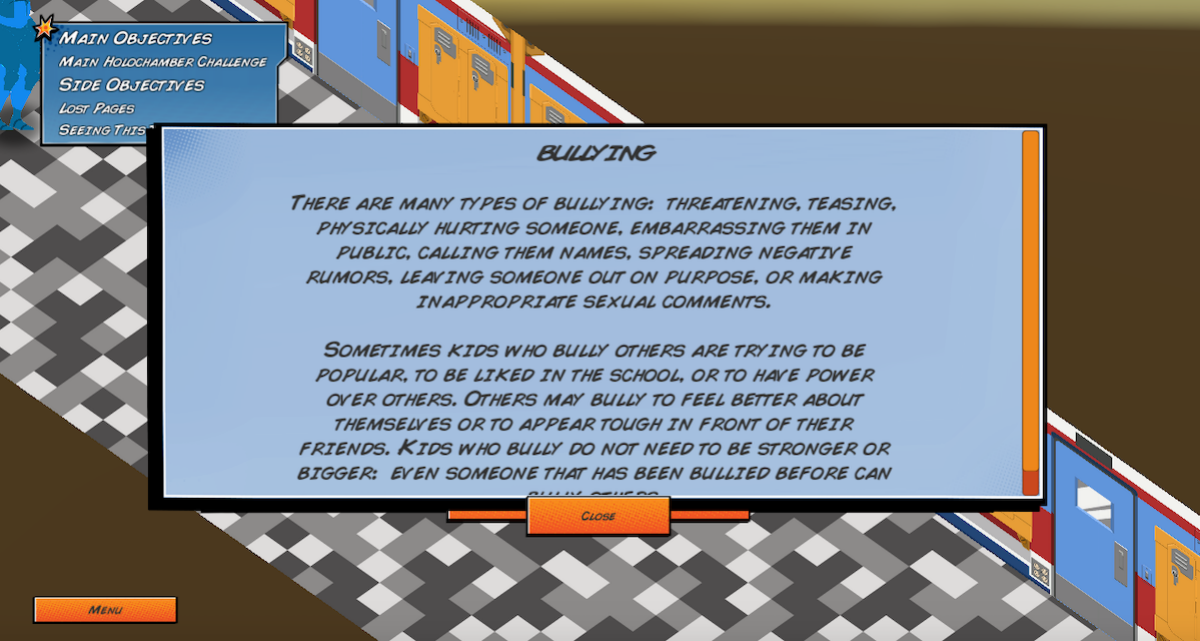
Virtual notebook page about bullying.

**Figure 8:**
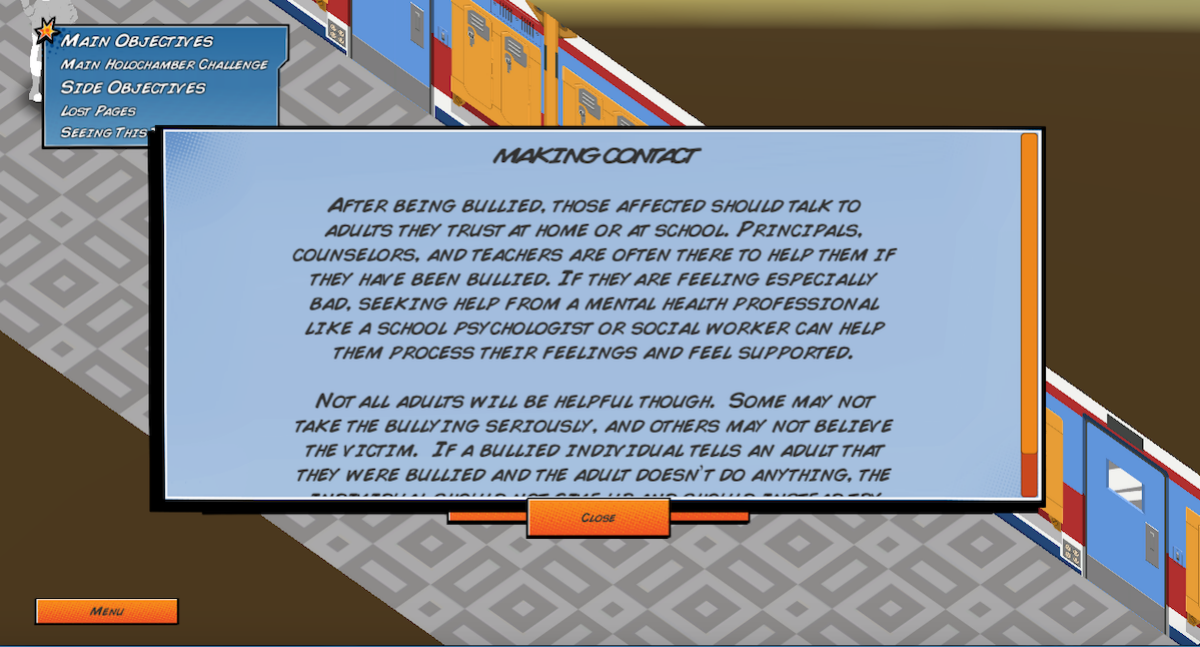
Virtual notebook page about the importance of making contact after being bullied.

Elements of these theories are embedded in the game in unique ways. First, the game encourages help-seeking behaviors by having players create a team with other nonplayable characters and to pair a lonely nonplayable character with an appropriate mentor (Figure 5). Second, the game encourages use of productive coping strategies through active listening and helping a nonplayable character overcome anger in a healthy way (Figure 6). Third, the game raises awareness of Web-based resources through collecting pages from a virtual notebook that contain information about external resources and bullying information (Figures 7 and 8).

#### Game Development

The game was developed in collaboration with Schell Games, an education and entertainment game development company located in Pittsburgh, Pennsylvania, United States. The intervention game is a role-playing game inspired by Japanese role-playing games, which typically involve exploring a space, talking to nonplayable characters, and fighting enemies through turn-based battles. The game runs standalone on desktops and laptops with Windows or Mac operating systems. Before implementation, we user-tested the intervention using think-aloud interviews with 3 SGMY, who found technical bugs and recommended password protection. Prior research shows that user testing with 3 participants results in finding at least 60% of major usability problems [90]. Schell Games revised the game accordingly before the implementation of the RCT.

#### Game Play

**Figure 9:**
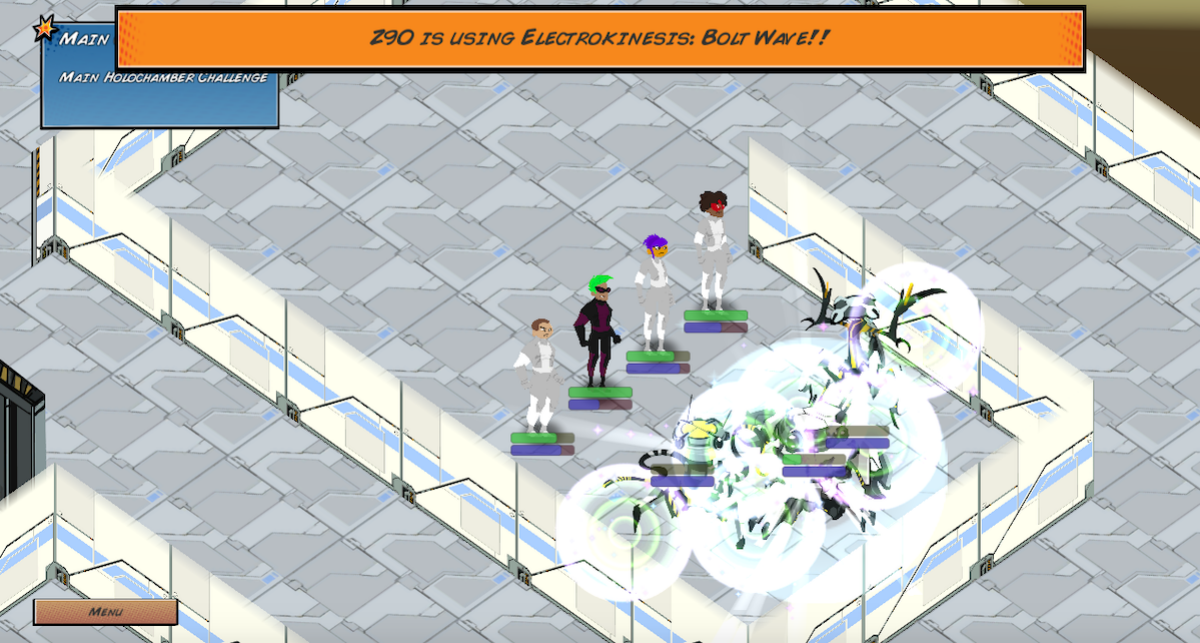
The player’s final mission: defeat the Holochamber Challenge with their teammates.

In the game, the player takes on the role of a Singular, a superhuman individual with special gifts, who is located in a school. The player is told that because of their uniqueness, Singulars face prejudice, often driven by fear and misunderstanding. After customizing their character, the player is tasked with finding a team to help them complete their final mission, which is to become a world-class superhero by defeating the robots in the Holochamber Challenge. The player then explores the school, talking to potential peers (ie, nonplayable characters) who can join their team. Some peers deal with problems related to bullying, confidence issues, and anger, which prevent them from performing properly or from wanting to join the player’s team. The player’s mini objectives are to do the following for each of their peers: (1) best identify the nonplayable characters’ problems, (2) find the best individuals or resources to help the nonplayable characters, and (3) help the nonplayable characters properly communicate or utilize their newfound resources. If the player is successful and finds the best way to help the nonplayable characters, then the nonplayable characters will join their team or help them by giving them an item or ability that will help them achieve success in the final Holochamber Challenge (Figure 9). After the player finishes the Holochamber Challenge, the events that took place are evaluated. For every nonplayable character that is successfully helped, the player is given a positive ending; for example, “Thanks to Invisibella’s mentoring, Violet Phantom eventually felt comfortable to reveal herself as a Singular. Despite past events, a majority of her other friends and family were supportive. Knowing she was not alone and had support helped her no longer feel afraid.” For every nonplayable character that was not successfully helped, the player is given a negative ending; for example, “Gigaton didn't learn to control his anger in this round. One day he snapped and turned on those bullying him. Though no one was seriously injured, the outburst resulted in Gigaton getting in trouble with the Principal.” The players are then encouraged to replay the game and given hints to help them receive positive endings. Each player can unlimitedly replay the game.

### Data Collection

We are collecting data in 2 distinct ways. First, we use self-administered surveys via REDCap, where participants completed surveys on their computer, tablet, or phone. Participants complete surveys at T1, T2, and T3 (see the section Study Population and Study Flow for more details). Surveys were created based on the Checklist for Reporting Results of Internet E-Surveys [91]. Second, we collect game play data from participants in the intervention condition. These game play data are transferred via a secure file-transfer-protocol system. Text files containing milestones achieved, time played, and player choices are automatically sent to a secure server housed at the University of Pittsburgh. Participants’ game play data are tracked using a unique identification number that does not rely on identifiable information (eg, IP addresses).

### Outcomes

#### Primary Outcomes

To answer our primary aim, we will assess the following primary feasibility outcomes: success of the implementation procedures used in our RCT, game demand, and game acceptability. Table 1 details our primary outcomes, assessments, and investigator-generated hypotheses.

Success of our implementation procedures are assessed using a variety of measures. We hypothesize the following: 240 participants will enroll in our trial and complete the first survey, greater than or equal to 80% of eligible participants will consent to participate in the study, 120 participants will be randomized to the intervention condition and 120 to the control condition, greater than or equal to 80% of enrolled participants will complete the T2 survey, greater than or equal to 80% of enrolled participants will complete the T3 survey, and greater than or equal to 75% of participants will complete all surveys. Testing the feasibility of our implementation procedures is essential to inform the design of our future larger-scale RCT.

**Table 1:** Primary outcomes, assessments, and hypotheses.

Primary outcome		Assessment	Hypothesis
**Implementation procedures**			
	Study population	Measured as the total number of participants who were consented and who completed T1^a^	240 participants
	Participation rate	Measured as the total number of people who agreed to participate in the study divided by the total number of people who were eligible to participate in the study	≥80%
	Number of randomized participants	Measured as the total number of participants randomized	240 participants
	Randomization success	Assessed by comparing intervention and control conditions across all demographic and potential confounding variables at baseline	No differences between intervention and control condition at baseline
	Retention rate for T2^b^	Measured as the total number of participants who completed T2 divided by the total number of participants enrolled in the study	≥80%
	Retention rate for T3^c^	Measured as the total number of participants who completed T3 divided by total number of participants enrolled in the study	≥80%
	Retention rate in T2 and T3	Measured as the total number of participants that completed both T2 and T3 surveys divided by the total number of participants enrolled in the study	≥75%
**Game demand**			
	Game download	Assessed on T2 and T3 surveys: did you download the game titled “Singularities”?—Yes; No; Unsure	≥80% selected “Yes”
	Any game play	Assessed on T2 and T3 surveys: did you play the game titled “Singularities”?—Yes; No; Unsure	≥80% selected “Yes”
	Any game play	Total number people who played the game based on game play data from the secure file-transfer-protocol system divided by total number of participants randomized to intervention condition	≥80% played
	Total time of game play	Assessed on T2 and T3 surveys: in the past month, about how long did you play the game “Singularities”?—I did not play the game; Less than 1 hour; 1 hour; 2 hours; 3 hours; 4 hours; 5 hours; 6 hours; 7 hours; 8 hours or more	≥75% selected 1 hour or greater
	Total time of game play	The number of hours the game was played based on game play data from the secure file-transfer-protocol system	≥75% played 1 hour or greater
**Game acceptability**			
	Gaming experience [92]	Assessed on T2 and T3 surveys: response options used a 5-point Likert scale (range: 0-4)—Not at all, Slightly, Moderately, Fairly, Extremely; Question stem was “Please indicate how you felt while playing the game for each of the questions below”; *Competence subscale* —I felt skillful, I felt competent, I was good at it, I felt successful, I was fast at reaching the game’s targets; *Sensory and imaginative immersion subscale* —I was interested in the game’s story, it was aesthetically pleasing, I felt imaginative, I felt that I could explore things, I found it impressive, It felt like a rich experience; *Flow subscale* —I was fully occupied with the game, I forgot everything around me, I lost track of time, I was deeply concentrated in the game, I lost connection with the outside world; *Tension and annoyance subscale* (reverse coded)—I felt annoyed, I felt irritable, I felt frustrated; *Negative affect subscale* (reverse coded)—It gave me a bad mood, I thought about other things, I found it tiresome, I felt bored; *Positive affect subscale* —I felt content, I thought it was fun, I felt happy, I felt good, I enjoyed it	Mean scores ≥2 for each subscale separately
	Desire to play game again	Assessed on T2 and T3 surveys: I would like to play this game again—Strongly Disagree; Disagree; Neutral; Agree; Strongly Agree	≥75% of intervention condition participants selected “Agree” or “Strongly Agree”
	Likelihood to recommend game to friends	Assessed on T2 and T3 surveys: how likely would you be to recommend that your friends play this game?—Definitely; Very Probably; Probably; Possibly; Probably Not; Definitely Not	≥75% of intervention condition participants selected “Definitely,” “Very Probably,” or “Probably”

^a^T1: baseline.

^b^T2: first follow-up.

^c^T3: final follow-up.

Game demand is assessed among the intervention condition participants in 2 ways, via downloading and playing the game. Download of the game is assessed via self-reported surveys (T2 and T3). We hypothesize ≥80% of intervention condition participants will download the game. Game play is assessed via self-reported surveys (T2 and T3) and game play data. We hypothesize ≥80% of intervention condition participants will play the game and ≥75% of intervention participants will play the game for 1 hour or greater.

Game acceptability among the intervention condition participants is assessed via self-reported surveys at T2 and T3. If participants reported playing the game, we ask about their overall impressions of the game using the Gaming Experience Questionnaire. The Gaming Experience Questionnaire is a multidimensional scale that assesses the following domains about the participants’ feelings and thoughts during the game: competence, sensory and imaginative immersion, game flow, game tension and annoyance (reverse coded), negative affect (reverse coded), and positive affect. We hypothesize that the mean score for each subscale will be greater than 2, representing a moderately good gaming experience. In addition, we ask participants if they would be interested in playing the game again, and whether they would recommend the game to their friends. We hypothesize ≥75% of participants will “agree” or “strongly agree” that they would like to play the game again, and ≥75% would “definitely,” “very probably,” or “probably” recommend the game to their friends.

#### Secondary Outcomes

To answer our secondary aim, we will measure the following short-term outcomes: help-seeking intentions, self-efficacy, and behaviors; productive coping strategy usage and coping flexibility; and knowledge and use of Web-based resources. All secondary outcomes are assessed via self-reported surveys at all time points. Table 2 details our secondary outcomes, items, response options, and coding procedures.

Help-seeking intentions are assessed using adapted version of the General Help Seeking Questionnaire [93]. In addition, 2 different sets of questions assess how likely participants are to seek help from a variety of sources about (1) emotional problems and (2) suicidal ideation. Help sources include a wide variety of people (eg, doctor and counselor) and places (eg, websites).

We adapted the original items by adding “Phone or text/chat help line (such as the National Suicide Prevention Lifeline or the Trevor Project)” and “teacher.” We will create 2 separate overall mean scores for how likely participants are to reach out concerning (1) emotional problems and (2) suicidal ideation. In addition, we will create a score for each help source individually. Higher mean scores indicate greater likelihood of seeking help from these sources.

Help-seeking self-efficacy is assessed using 2 subscales from Bandura’s Multidimensional Scales of Perceived Self Efficacy [94,95]: the enlisting social resources subscale and the enlisting parental and community support subscale. Each subscale has 4 items, which are used to calculate an overall mean score measuring how well participants think they can get support from different sources (eg, “How well can you get a friend to help you when you have social problems” and “How well can you get your parents to take part in school activities”).

Help-seeking behaviors are assessed using items adapted from the Help-Seeking Behaviors Scale [96]. We adapted the scale to include the wide variety of help sources used in the aforementioned help-seeking intention scales. Therefore, we created 8 different subscales (1 for each help source) assessed with 3 items each (eg, “In the past month, how often have you asked a teacher for help?”). Each subscale will be analyzed separately.

Coping skill usage is assessed using the Adolescent Coping Scale Second Edition Short Form [88]. This scale uses 18 items measuring 2 different dimensions: productive (problem solving) coping and nonproductive (passive avoidant) coping. We hypothesize that the game-based intervention (vs the control) will increase productive coping and decrease nonproductive coping.

Coping flexibility is assessed using the Coping Flexibility Scale [97], including 2 separate subscales: evaluation coping and adaptive coping. The evaluation coping subscale uses 5 items to assess how well a person monitors and evaluates coping outcomes (eg, “I am aware of how successful or unsuccessful my attempts to cope with stress have been.”). The adaptive coping subscale uses 5 items to assess how well a person uses an alternative coping strategy to produce a desirable outcome (eg, “When a stressful situation has not improved, I try to think of other ways to cope with it”). Each subscale will be examined separately.

Knowledge and use of Web-based resources are measured using 2 different scales developed by the investigative team. We created a list of SGM-inclusive resources that match what was provided in the game-based intervention and control materials, such as the Trevor Project. Use of Web-based resources is measured for the past month. We will create 1 summary score for knowledge and 1 for use; each summary score adds together all “yes” response options.

**Table 2:** Secondary outcomes, items, response options, and coding procedures.

Secondary outcome		Items	Response options	Coding procedure
**Help-seeking intentions, self-efficacy, and behaviors**				
	Help-seeking intentions for personal or emotional problems, adapted from [93]	If you were having a personal or emotional problem, how likely is that you would seek help from the following people?—parent or guardian; other relative or family member; teacher; intimate partner (such as girlfriend or boyfriend); friend (someone not related to you); mental health professional (such as a psychologist, social worker, counselor); phone or text/chat help line (such as the National Suicide Prevention Lifeline or the Trevor Project); website help resources (such as StopBullying.gov); doctor or primary care provider; minister or religious leader (eg, priest, rabbi, chaplain); I would not seek help from anyone; I would seek help from another person/place not listed above	7-point Likert scale, ranging from “extremely unlikely” (1) to “extremely likely” (7)	Mean score averaged across all items and for each item individually
	Help-seeking intentions for suicidality, adapted from [93]	If you were experiencing suicidal thoughts, how likely is that you would seek help from the following people?—parent or guardian; other relative or family member; teacher; intimate partner (such as girlfriend or boyfriend); friend (someone not related to you); mental health professional (such as a psychologist, social worker, counselor); phone or text/chat help line (such as the National Suicide Prevention Lifeline or the Trevor Project); website help resources (such as StopBullying.gov); doctor or primary care provider; minister or religious leader (eg, priest, rabbi, and chaplain); I would not seek help from anyone; I would seek help from another person/place not listed above	7-point Likert scale, ranging from “extremely unlikely” (1) to “extremely likely” (7)	Mean score averaged across all items and for each item individually
	Help-seeking self-efficacy [94,95]	Please rate how certain you are that you can do each of the things described; *Enlisting social resources subscale* —how well can you get teachers to help you when you get stuck on schoolwork? how well can you get another student to help you when you get stuck on schoolwork? how well can you get adults to help you when you have social problems? how well can you get a friend to help you when you have social problems?; *Enlisting parental and community support subscale* —how much can you get your parent(s) to help you with a problem? how well can you get your brother(s) and sister(s) to help you with a problem? how well can you get your parents to take part in school activities? how well can you get people outside the school to take an interest in your school (community groups, churches)?	7-point Likert scale, ranging from “not at all well” (1) to “very well” (7)	Mean score for each subscale separately
	Help-seeking behaviors, adapted from [96]	In the past month, how often have you: *Parent and guardian help-seeking subscale* —asked your parents/guardians for help? talked to your parents/guardians about personal problems? talked to your parents/guardians about problems at school?; *Relative/family help-seeking subscale* —asked a relative/family member for help? talked to a relative/family member about personal problems? talked to a relative/family member about problems at school?; *Teacher help-seeking subscale* —asked a teacher for help? talked to a teacher about personal problems? talked to a teacher about problems at school?; *Friend help-seeking subscale* —asked a friend for help? talked to a friend about personal problems? talked to a friend about problems at school?; *Mental health provider help-seeking subscale* —asked a mental health professional (such as a psychologist, social worker, counselor) for help? talked to a mental health professional about personal problems? talked to a mental health professional about problems at school?; *Help line help-seeking subscale* —asked a person from a phone or text/chat help line (such as the National Suicide Prevention Lifeline or the Trevor Project) for help? talked to a person from a phone or text/chat help line about personal problems? talked to a person from a phone or text/chat help line about problems at school?; *Doctor help-seeking subscale* —asked a doctor or nurse for help? talked to a doctor or nurse about personal problems? talked to a doctor or nurse about problems at school?	5-point Likert scale: Never; Rarely; Occasionally; A moderate amount; A great deal	Mean score for each subscale separately
**Coping strategies and flexibility**				
	Coping skill usage [88]	Assessed using the Short form of the Adolescent Coping Scale Second Edition Short Form (ACS-2) [88]. For proprietary reasons, we do not list specific items. *Productive (problem-solving) coping subscale* contains 10 items; *Nonproductive (passive avoidant) coping subscale* contains 8 items (reverse coded)	5-point Likert Scale, ranging from “never” (1) to “very often” (5)	Mean score for each subscale separately
	Coping flexibility [97]	Please indicate how these situations apply to you by choosing one of the following for each situation: *Evaluation coping subscale* —I only use certain ways to cope with stress (reverse-coded); I am aware of how successful or unsuccessful my attempts to cope with stress have been; I fail to notice when I have been unable to cope with stress (reverse-coded); if I feel that I have failed to cope with stress, I change the way in which I deal with stress; after coping with stress, I think about how well my ways of coping with stress worked or did not work; *Adaptive coping subscale* —when a stressful situation has not improved, I try to think of other ways to cope with it; when stressed, I use several ways to cope and make the situation better; when I haven’t coped with a stressful situation well, I use other ways to cope with that situation; if a stressful situation has not improved, I use other ways to cope with that situation; if I have failed to cope with stress, I think of other ways to cope	4-point Likert Scale: Not applicable; Somewhat applicable; Applicable; Very applicable	Mean score for each subscale separately
**Knowledge and use of Web-based resources**				
	Knowledge of Web-based resources	Have you heard of any of these websites?—The Trevor Project; It Gets Better; GLAAD; Accredited Schools online; Teen Line; GSA^a^ Network. (Links to each website were provided for reference.)	Yes; No; Unsure	Summary score that adds together all “yes” response options
	Use of Web-based resources	Have you visited any of these websites in the past month?—The Trevor Project; It Gets Better; GLAAD; Accredited Schools online; Teen Line; GSA^a^ Network. (Links to each website were provided for reference.)	Yes; No; Unsure	Summary score that adds together all “yes” response options

^a^GSA: gay-straight alliance or gender and sexuality alliance.

#### Tertiary Outcomes

To answer our tertiary aim, we will measure the following long-term outcomes: bullying and cyberbullying victimization, loneliness, mental health issues, substance use, and internalized SGM stigma. All tertiary outcomes are assessed via self-reported surveys at T1, T2, and T3. Table 3 details our tertiary outcomes, items, response options, and coding procedures.

Bullying victimization is measured using 2 different scales, 1 for traditional face-to-face bullying and 1 for cyberbullying. We assess past-month bullying victimization using an adapted version of the University of Illinois Victimization Scale [98,99], which consists of 6 items assessing the number of times respondents experienced harassment in the past month. In addition, 4 response options range from “never” to “7 or more.” These response options are assigned a value from 1 to 4, and a mean score is calculated, with a higher mean score indicating greater bullying victimization. We assess past-month cyberbullying victimization using 4 items adapted from an internally consistent cyberbullying perpetration scale [100]. Response options use a 5-point Likert scale including “not sure,” “never,” “rarely,” “occasionally,” and “often,” which we will recode to a 4-point scale, where “never” equals 1 and “often” equals 4. We will average scores across all items, with a higher mean score indicating greater cyberbullying victimization.

Loneliness is measured via Robert’s Version of the University of California, Los Angeles Loneliness Scale [101], a validated measure of loneliness. Overall, 8 items (eg, “I feel isolated from others”) are used to calculate a mean score for how lonely participants felt.

Mental health issues include stress, anxiety, depression, and suicidality. Past-month stress is assessed using the Perceived Stress Scale [102,103], which contains 10 items used to calculate a mean score. Past-week anxiety symptoms are assessed using the Severity Measure for Generalized Anxiety Disorder—Child Age 11-17 [104,105], which contains 10 items used to calculate a mean score. Past-week depressive symptoms are assessed using the Patient Health Questionnaire-9 for children aged from 11 to 17 years [106], which contains 9 items used to calculate a mean score. Suicidality is assessed with 3 questions adapted from the 2017 YRBS with items measuring suicidal thoughts, plans, and attempts. Although the YRBS questions measured past year suicidality, we adapted them to assess for suicidality in the past month. Each of these 3 suicidality items will be modeled separately as dichotomous (presence vs absence) variables.

Substance use includes alcohol, cigarette, electronic cigarette, and marijuana use. All items are assessed based on the YRBS and adapted to measure past-month use. Alcohol use is assessed via 2 questions: 1 measuring the frequency of past-month alcohol use and 1 item assessing frequency of binge drinking (having 5 or more drinks in a row). Similar to prior research [7], each item will be modeled as continuous variable representing the number of days used. Cigarette smoking is assessed via 2 items measuring past-month number of days smoked and average quantity smoked per day. Cigarette smoking will be modeled continuously by multiplying smoking frequency by quantity, similar to prior research [10]. Electronic cigarette smoking and marijuana use are both assessed using single items that measure past-month frequency of use. Each will be modeled as a continuous variable representing the number of days used. For all substance use items, we will use midpoints for categories with a range (eg, “1 or 2 days” will be coded as 1.5).

Internalized SGM stigma is measured using 2 different scales. To assess internalized gender minority stigma, we use the Transgender Identity Survey [107,108], which consists of 26 items that are divided into 4 different subscales: pride (reverse coded), passing, alienation, and shame. We adapted this scale by adding the term “nonbinary” to each item. To assess internalized sexual minority stigma, we adapted the Transgender Identity Survey [107,108] items by replacing “transgender or nonbinary” with “lesbian, gay, bisexual, or queer”; otherwise, the subscales, response options, and scoring are identical.

**Table 3:** Tertiary outcomes, items, response options, and coding procedures.

Tertiary outcome		Items	Response options	Coding procedure
**Bullying and cyberbullying victimization**				
	Bullying victimization [98,99]	How many times did these things happen to you in the last 30 days?—other students picked on me; other students called me names; I got hit and pushed by other students; I was threatened by other students; students spread rumors or told lies about me; I was excluded or kept out of a group of friends on purpose.	Never; 1 or 2 times; 3 or 4 times; 5 or 6 times; 7 or more times	Mean score
	Cyberbullying victimization, adapted from [100]	How often did the following things happen to you in the last month?—someone made rude comments about me online; someone spread rumors about me online, whether they were true or not; someone made aggressive or threatening comments to me online; someone sent a text message that said rude or mean things about me	Not sure; Never; Rarely; Occasionally; Often	Mean score
**Loneliness**				
	Loneliness [101]	Indicate how often each of the statements below is descriptive of you—I feel in tune with the people around me (reverse coded); I lack companionship; I do not feel alone (reverse coded); I feel part of a group of friends (reverse coded); I am no longer close to anyone; I feel left out; I feel isolated from others; I can find companionship when I want it (reverse coded)	4-point Likert scale: Never; Rarely; Sometimes; Often	Mean score
**Mental health issues**				
	Stress [103,109]	For each item, mark the description that best represents how often you have felt or thought that way during the past month—Been upset because of something that happened unexpectedly; Felt that you were unable to control the important things in your life; Felt nervous and ― “stressed”; Felt confident about your ability to handle your personal problems; Felt that things were going your way; Found that you could not cope with all the things that you had to do; Been able to control irritations in your life; Felt that you were on top of things; Been angered because of things that were outside of your control; Felt difficulties were piling up so high that you could not overcome them	5-point Likert scale: Never; Almost never; Sometimes; Fairly often; Very often	Mean score
	Anxiety [104,105]	During the past 7 days, I have: Felt moments of sudden terror, fear, or fright; Felt anxious, worried, or nervous; Had thoughts of bad things happening such as family tragedy, ill health, loss of a job, or accidents; Felt a racing heart, sweaty, trouble breathing, faint, or shaky; Felt tense muscles, felt on edge or restless, or had trouble relaxing or trouble sleeping; Avoided, or did not approach or enter situations about which I worry; Left situations early or participated only minimally because of worries; Spent lots of time making decisions, putting off making decisions, or preparing situations, because of worries; Sought reassurance from others because of worries; Needed help to cope with anxiety (eg, alcohol or medication, superstitious objects, or other people)	5-point Likert scale: Never; Occasionally; Half the time; Most of the time; All of the time	Mean score
	Depression [106]	How often have you been bothered by each of the following symptoms during the past 7 days?—Feeling down, depressed, irritable, or hopeless; Little interest or pleasure in doing things; Trouble falling asleep, staying asleep, or sleeping too much; Poor appetite, weight loss, or overeating; Feeling tired, or having little energy; Feeling bad about yourself—or feeling that you are a failure, or that you have let yourself or your family down; Trouble concentrating on things like school work, reading, or watching TV; Moving or speaking so slowly that other people have noticed; Or the opposite—being so fidgety or restless that you were moving around a lot more than usual; Thoughts that you would be better off dead, or of hurting yourself in some way	4-point Likert scale: Not at all; Several days; More than half the days; Nearly every day	Mean score
	Suicidal ideation, adapted from [110]	During the past month, did you ever seriously consider attempting suicide?	Yes; No	Dichotomously as yes or no
	Suicide plan, adapted from [110]	During the past month, did you make a plan about how you would attempt suicide?	Yes; No	Dichotomously as yes or no
	Suicide attempt, adapted from [110]	During the past month, how many times did you actually attempt suicide?	0 times; 1 time; 2 or 3 times; 4 or 5 times; 6 or more times	Dichotomously as yes or no, similar to prior research [6]
**Substance use**				
	Alcohol use [110]	During the past 30 days, on how many days did you have at least one drink of alcohol?	0 days; 1 or 2 days; 3 to 5 days; 6 to 9 days; 10 to 19 days; 20 to 29 days; All 30 days	Count variable using midpoints (eg, “1 or 2 days” equals 1.5), similar to prior research [7]
	Binge alcohol use [111]	During the past 30 days, on how many days did you have 5 or more drinks of alcohol in a row (within a couple of hours)?	0 days; 1 day; 2 days; 3 to 5 days; 6 to 9 days; 10 to 19 days; 20 or more days	Count variable using midpoints (eg, “1 or 2 days” equals 1.5), similar to prior research [7]
	Cigarette smoking [110]	During the past 30 days, on how many days did you smoke cigarettes? During the past 30 days, on the days you smoked, how many cigarettes did you smoke per day?	For the question pertaining to number of days: 0 days; 1 or 2 days; 3 to 5 days; 6 to 9 days; 10 to 19 days; 20 to 29 days; All 30 days. For the question pertaining to number of cigarettes: I did not smoke cigarettes during the past 30 days; Less than 1 cigarette per day; 1 cigarette per day; 2 to 5 cigarettes per day; 6 to 10 cigarettes per day; 11 to 20 cigarettes per day; More than 20 cigarettes per day	Continuously by multiplying frequency by quantity, similar to prior research [10]
	Electronic cigarette use [110]	During the past 30 days, on how many days did you use an electronic vapor product?	0 days; 1 or 2 days; 3 to 5 days; 6 to 9 days; 10 to 19 days; 20 to 29 days; All 30 days	Count variable using midpoints (eg, “1 or 2 days” equals 1.5)
	Marijuana use [110]	During the past 30 days, how many times did you use marijuana? (Marijuana is also called grass, pot, or weed.)	0 times; 1 or 2 times; 3 to 9 times; 10 to 19 times; 20 to 39 times; 40 or more times	Count variable using midpoints (eg, “1 or 2 days” equals 1.5)
**Internalized sexual and gender minority stigma**				
	Internalized gender minority stigma, adapted from [107,108]	Pride subscale contains 8 items (reverse coded); Passing subscale contains 7 items; Alienation subscale contains 3 items; Shame subscale contains 8 items; To obtain the specific scale items, please contact Robert WS Coulter and Walter O Bockting	7-point Likert Scale: Strongly disagree; Disagree; Somewhat disagree; Neither agree nor disagree; Somewhat agree; Agree; Strongly agree	Mean score for each subscale separately
	Internalized Sexual Minority Stigma, adapted from [107,108]	Pride subscale contains 8 items (reverse coded); Passing subscale contains 7 items; Alienation subscale contains 3 items; Shame subscale contains 8 items; To obtain the specific scale items, please contact Robert WS Coulter and Walter O Bockting	7-point Likert Scale: Strongly Disagree; Disagree; Somewhat Disagree; Neither Agree nor Disagree; Somewhat Agree; Agree; Strongly Agree	Mean score for each subscale separately

#### Exploratory Outcomes

To answer our exploratory aim, we assess several outcomes meant to better our understanding of participants’ responses to the game and how to improve the game in the future. Our exploratory aim concerns implementation procedures, intervention integration, and intervention adaptation and expansion outcomes. Table 4 details our exploratory outcomes, research questions, and assessments.

Multiple implementation procedures will be explored to inform our future research. These include the following: how long it takes to enroll participants on the internet, which venues participants were recruited from, how long it takes participants to complete the surveys, how many participants complete the game, and which game milestones are achieved by participants. These data are obtained from multiple sources, including the self-reported surveys in REDCap and the game play data.

Implementation integration—or how well the game fits into the participants’ lives—will be assessed in a variety of ways. We will examine how many participants were excluded from our study based on not having access to a computer or not having an email address. We will examine if the participants had any problems downloading or playing game and how easy it was for them to participate in the RCT. We will also explore if participants had issues with game security, privacy, or interference. These outcomes are tracked using participants’ responses to the screening questionnaire, T2 survey, and T3 surveys, as well as from participants’ correspondences with the project email address.

Adaptation and expansion of the game intervention will be assessed via the T2 and T3 surveys. We will ask participants if they prefer to play the game on a different format (eg, on their phones), their favorite and least favorite parts of the game, how they would change and improve the game, and whether they think other SGMY would be interested in the game. Finally, we will explore whether the responses to the Gaming Experience Questionnaire [92] subscale scores differed by participants’ gender, sexual orientation, race, ethnicity, and age.

**Table 4:** Exploratory outcomes, research questions, and assessments.

Exploratory outcome		Exploratory question	Assessment
**Implementation procedures**			
	Enrollment period	How long does it take to get 240 people to enroll in the study?	Measured as number of months between enrollment of first and last participant.
	Recruitment venues	How many people were recruited from which venue?	Assessed using unique links that track the number of clicks on each advertisement.
	Survey completion time	How many days before participants completed each survey?	Measured as the number of days between the first survey invitation was sent to the participant and the day they completed the survey.
	Game completion	How many intervention condition participants completed the game?	Assessed via game play data from the secure file-transfer-protocol (FTP) system as well as T2^a^ and T3^b^ surveys: Did you complete the game? Yes; No; Unsure
	Game milestones	What milestones did the participants achieve in the game?	Assessed via game play data from the secure file-transfer-protocol (FTP) system.
**Integration**			
	Computer access	How many youths were excluded because they were without a personal computer or Mac?	Assessed via the screening questionnaire: Do you have a laptop or desktop computer (either personal computer or Mac) where you can download games? Yes; No; Unsure
	Email address access	How many youths were excluded because they were without an email address?	Assessed via the screening questionnaire: do you have an email address? Yes; No
	Ease of download	How easily was the game downloaded without contacting our research coordinator?	Assessed via the number of emails to our project email address.
	Personal computer versus Mac use	What participants used personal computer and Mac computers to download the game?	Assessed as percentages via the game download materials in REDCap.
	Ease of participation	How many times was the research staff contacted by participants with questions about surveys or intervention materials?	Assessed via the number and types of emails to our project email address.
	Game problems	How many problems and what types of problems did participants encounter with the game?	Assessed via T2 and T3 surveys: Did you have any problems in the game?—Yes; No; If yes, please describe; Open-ended text box
	Game security	How important was the use of password protection in the game?	Assessed via T2 and T3 surveys: How important was it to have the game be protected by a password?—Very Important; Important; Moderately Important; Slightly Important; Not Important
	Game privacy	How safe did our participants feel playing the game?	Assessed via T2 and T3 surveys: How often were you concerned about other people seeing you play the game?—Always; Very Often; Sometimes; Rarely; Never
	Game interference	Did the gaming intervention interfere with participants’ regular activities?	Assessed via T2 and T3 surveys: During the past 30 days, how many times did playing the game interfere with school, work, or other responsibilities (like being late, missing school, or making it hard to concentrate)—Never; 1 time; 2 times; 3 times; 4 times; 5 or more times
**Adaptation and expansion**			
	Future gaming platforms	Would our participants like to play the game on other platforms, such as phone?	Assessed via T2 and T3 surveys: In the future where would you like to play this game? My computer only; My phone only; Both my computer and phone
	Gaming experience [92] by demographics	Were there certain groups that had better or worse experiences with the game?	We will examine whether the Gaming Experience Questionnaire [92] subscale scores (assessed via T2 and T3 surveys) differed by participants’ gender, sexual orientation, race, ethnicity, and age.
	Future appeal to sexual and gender minority youth	Will other sexual and gender minority youth enjoy the game?	Assessed via T2 and T3 surveys: Do you think other LGBTQ people your age would like to play this game? Definitely; Very Probably; Probably; Possibly; Probably Not; Definitely Not
	Recommended game changes	What would participants change about the game?	Assessed via T2 and T3 surveys: What would you change about the game? Open-ended text box
	Favorite parts of game	What did participants like best about the game?	Assessed via T2 and T3 surveys: What were your favorite parts of the game? Open-ended text box
	Least favorite arts of game	What did participants like least about the game?	Assessed via T2 and T3 surveys: What were your least favorite parts of the game? Open-ended text box
	Recommended game improvements	How would participants improve the game?	Assessed via T2 and T3 surveys: How would you improve the game? Open-ended text box

^a^T2: first follow-up.

^b^T3: final follow-up.

### Demographics and Potential Confounders

Demographic variables include age, grade in school, race, ethnicity, parent’s highest education level, and eligibility for free or reduced-price lunch at school. We also measure gender using the 2-step process assessing current gender identity and sex assigned at birth [112]. Moreover, 2 items assess gender expression. We measure sexual orientation using 3 questions assessing sexual attraction, behavior, and identity.

In addition to demographic variables, we assess variables that may confound the relationship between the intervention and our outcomes. Structural stigma is assessed by collecting participant’s residential zip code. Each zip code will then be given a state-level structural stigma score using Hatzenbuehler scale [113], based on density of same-sex couples, inclusive policies (eg, employee nondiscrimination policies), public opinion, and percentage of high schools with gay-straight alliance (also known as a gender and sexuality alliance; GSA). We have participants self-report whether or not their school had a GSA. We assess the participants’ overall school environment with 5 questions from the California School Climate Survey. We assess participants’ level of perceived social support using the Multidimensional Scale of Perceived Social Support, measuring support from family and friends [114]. Finally, we measure level of “outness” regarding participant’s sexual orientation and gender identity.

### Statistical Analysis

For the measures of implementation procedures, demand, acceptability, integration, and adaptation and expansion (ie, primary and exploratory outcomes), we will report results using descriptive statistics (ie, percentages and frequencies for categorical variables or means and SDs for continuous variables) along with 95% CIs. We will compare our results against our a priori benchmarks (see Table 1). For the limited-efficacy outcomes (ie, secondary and tertiary outcomes), we will use repeated measures (ie, multilevel) statistical models using linear or logistic regression, depending on the distribution of the outcomes. To examine whether there are greater improvements over time in the intervention versus control conditions, we will test the interaction term of time by condition, wherein we will focus on the point estimates and 95% CIs. We will conduct our primary analyses as an intent-to-treat analysis, wherein everyone in the intervention condition is treated equally. As an additional exploratory exercise, we will conduct an analysis wherein we assess whether intervention effects were stronger with more intensive uptake of the game intervention. For these analyses, instead of a binary intervention indicator variable (1.0 for intervention vs 0.0 for control), we will use an intensity-adjusted intervention variable. The intensity-adjusted intervention variable will be coded as 1.0 for those who complete the game, 0.5 for those who play the game, and 0.0 for those who never played the game or were in the control condition. All analyses will be completed in Stata 15.0 (StataCorp LLC, College Station, Texas), and significance will be set as at *P*<.05.

### Qualitative Analysis

For the open-ended questions, we will conduct qualitative data analysis. First, a group of investigators will read all of the participants’ responses and inductively identify the main themes for each item. Second, using these themes, we will develop a codebook with code names and definitions. Finally, 2 independent coders will code the qualitative data using Dedoose. If the 2 coders cannot reach consensus, a third coder will resolve any disagreements.

### Sample Size and Power Calculation

This is a feasibility study, which is primarily being conducted to inform a larger RCT. As such, we powered this study based on our primary outcomes [74,115]. For our primary outcomes regarding the success of implementation procedures (eg, retention rate at T2), assuming 240 participants and 5% type 1 error rate, we will be able to estimate 95% CI width of no more than 0.13. For game demand among 120 intervention condition participants, we will be able to estimate 95% CI width of no more than 0.18. For our secondary and tertiary outcomes, we are primarily interested in effect size and CI estimation; we are not necessarily interested in finding statistically significant effects [74,115]. Estimations of the effect size and CI width will help us power a future, larger RCT.

### Ethics Statement

All study procedures were approved by the Human Research Protection Office at the University of Pittsburgh. To protect participants from having to reveal their sexual or gender minority identities to their caregivers, thereby potentially putting them in harm’s way, we received a waiver of parental consent [76]. This allowed participants to self-consent. We did not collect personally identifying information other than email, phone, and zip code. Incentives, in the form of either Apple or Google electronic gift cards, are sent via email. This study was covered by a Certificate of Confidentiality from the National Institutes of Health.

## Results

### Enrollment and Randomization

**Figure 10:**
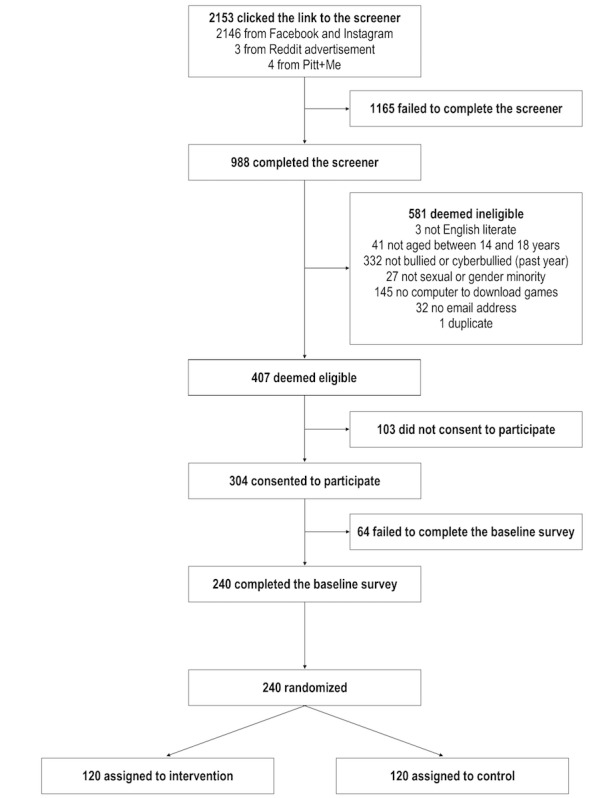
Consolidated Standards of Reporting Trials (CONSORT) flow diagram.

Overall, 2153 individuals clicked the link to the screening questionnaire (Figure 10). In total, 988 individuals completed the screening questionnaire, of which 407 individuals met all eligibility criteria. Overall, 304 individuals consented to participate, and 240 participants completed the T1 survey and were randomized. Of those who were eligible, 59.0% (240/407) were enrolled into the study. Half of the enrolled participants (n=120) were randomized into the intervention condition and half (n=120) into the control condition. All 240 participants were enrolled into the RCT between April 2018 and July 2018. The final surveys (T3) will be completed in November 2018.

### Sample Demographics

**Figure 11:**
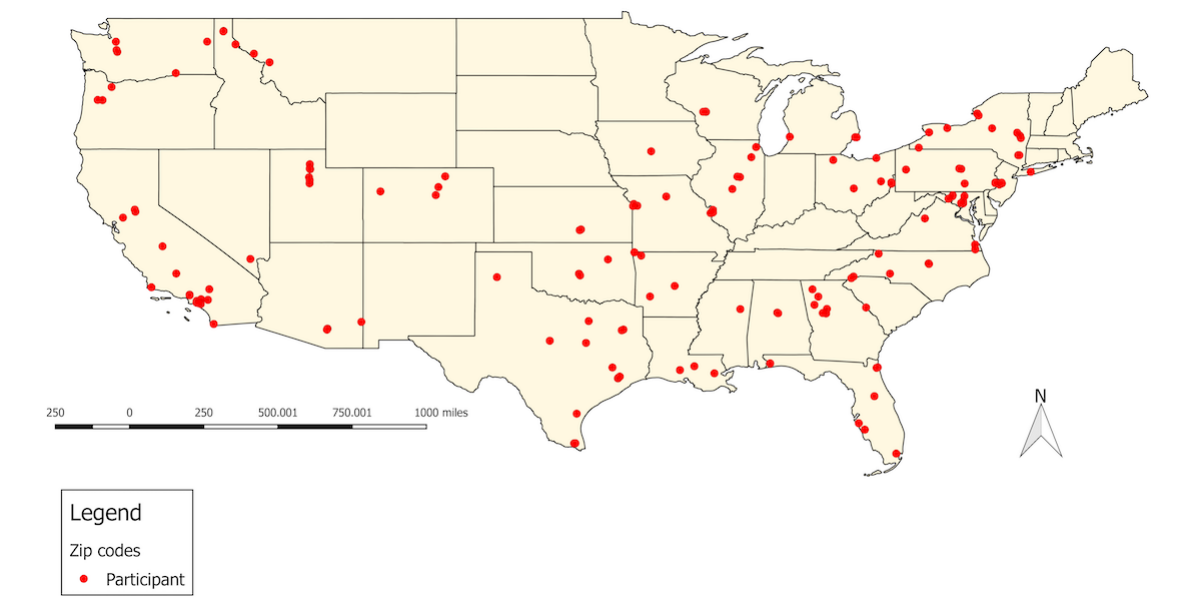
Geographic distribution of participants in the United States of America.

At baseline, there were no significant differences in demographic characteristics between intervention and control condition participants (Table 5). Overall, 52.1% (125/240) of participants identified as gay or lesbian, 26.7% (64/240) as bisexual, 24.2% (58/240) as queer, 11.7% (28/240) as another nonheterosexual identity, 4.6% (11/240) as unsure, and 2.1% (5/240) as heterosexual (all of whom were gender minorities). Nearly half (47.1%; 113/240) of participants were a gender minority, 36.7% (88/240) were cisgender boys, and 16.3% (39/240) were cisgender girls. Among the 113 gender minority participants, 54.9% (62/113) identified as a transgender boy, 33.6% (38/113) as nonbinary, 18.6% (21/113) as a boy, 17.7% (20/113) as genderqueer, 6.2% (7/113) as a transgender girl, 5.3% (6/113) as a girl, and 5.3% (6/113) as another identity (data not shown; participants could select multiple options). Overall, 81.3% (195/240) of participants identified as white, 20.4% (49/240) as Hispanic/Latinx, 10.8% (26/240) as black or African American, 7.5% (18/240) as American Indian or Alaska Native, and 5.8% (14/240) as Asian. Moreover, 36.7% (88/240) of participants were eligible for free or reduced-price lunch, and 52.9% (127/240) had a parent/guardian with a college degree. Figure 11 shows the geographic distribution of participants across the United States of America. In total, participants lived in 30 states.

**Table 5:** Baseline demographic characteristics for the total sample and by intervention condition (N=240).

Demographic characteristics		Total (N=240), n (%)	Condition		
			Intervention (n=120), n (%)	Control (n=120), n (%)	*P* value^a^
**Sexual identity^b^**					
	Gay or lesbian	125 (52)	62 (52)	63 (53)	.89
	Bisexual	64 (27)	32 (27)	32 (27)	>.99
	Queer	58 (24)	33 (28)	25 (21)	.23
	Unsure	11 (5)	8 (7)	3 (3)	.12
	Another nonheterosexual identity	28 (12)	11 (9)	17 (14)	.23
	Heterosexual	5 (2)	3 (3)	2 (2)	>.99
**Gender**					.24
	Cisgender girls	39 (16)	18 (15)	21 (18)	
	Cisgender boys	88 (37)	39 (33)	49 (41)	
	Gender minority^c^	113 (47)	63 (53)	50 (42)	
**Age in years**					.98
	14	35 (15)	16 (13)	19 (16)	
	15	66 (28)	33 (28)	33 (28)	
	16	71 (30)	37 (31)	34 (28)	
	17	56 (23)	28 (23)	28 (23)	
	18	12 (5)	6 (5)	6 (5)	
**Race/ethnicity^b^**					
	White	195 (81)	96 (80)	99 (83)	.62
	Asian	14 (6)	5 (4)	9 (8)	.27
	American Indian or Alaska Native	18 (8)	6 (5)	12 (10)	.14
	Black or African American	26 (11)	15 (13)	11 (9)	.41
	Native Hawaiian or other Pacific Islander	5 (2)	3 (3)	2 (2)	>.99
	Hispanic or Latinx	49 (20)	28 (23)	21 (18)	.26
	Another race	21 (9)	9 (8)	12 (10)	.49
**Free or reduced-price lunch eligibility**					.98
	Yes	88 (37)	44 (37)	44 (37)	
	No	109 (45)	54 (45)	55 (46)	
	Unsure	43 (18)	22 (18)	21 (18)	
**Highest education level of parent(s) or guardian(s)**					.75
	Less than high school degree	22 (9)	10 (8)	12 (10)	
	High school degree	38 (16)	22 (18)	16 (13)	
	Some college	43 (18)	23 (19)	20 (17)	
	College degree	127 (53)	61 (51)	66 (55)	
	Unsure	10 (4)	4 (3)	6 (5)	

^a^Generally, *P* values were derived using chi-square test statistics; however, when expected cell sizes were less than 5, *P* values were derived using Fisher exact tests.

^b^Participants could select more than one option; therefore, the percentages may not add to 100% (N=240). *P* values were derived for each response option separately.

^c^Gender minority includes all participants whose gender identity did not match their assigned sex at birth.

## Discussion

### Principal Findings

In this pilot RCT, a game-based intervention, we successfully enrolled and randomized 240 SGMY participants over a 4-month period. Recruitment was most successful through Facebook and Instagram. Over half of the enrolled sample identified as gay or lesbian and nearly half were gender minority youth. There were no demographic differences between intervention and control conditions.

This study protocol offers several strengths and novel methods that can inform future intervention studies with SGMY. Our game intervention is one of the first Web-accessible programs targeting SGMY who are victims of bullying or cyberbullying. Furthermore, this study engages a younger cohort of individuals focusing on youth aged 14 to 18 years, as opposed to the typical focus of SGMY-relevant intervention research, which concentrates on those aged 18 years and older. This is also one of the first bullying prevention programs to address social and emotional learning (eg, coping strategies) with respect to bullying among SGMY utilizing a gaming format to attract participation and build retention. Finally, the internet-based distribution of the game intervention has the potential to reach far greater numbers of SGMY than traditional face-to-face interventions.

### Limitations

Despite the many strengths of this study protocol, it is not without limitations. Of particular concern is selection bias. Participants in our study were conveniently sampled via the internet. Participants were also required to have a computer with internet access and an email address. Therefore, our study results may not be generalizable to all SGMY (eg, those who are homelessness or have a low socioeconomic status). In addition, participants can only access the game intervention on desktop or laptop computers with Windows or Mac operating systems. In future iterations, we plan to make the game intervention compatible with smartphones, thereby increasing the accessibility and potential usability of the game. Although we used and adapted measures with strong psychometric properties, most of the scales were not validated specifically among SGMY. Finally, an 8-week follow-up period may be too brief to affect our identified long-term outcomes; however, we chose to measure these outcomes as they are important health outcomes to inform a longer-term intervention study.

### Conclusions

SGMY experience great disparities in bullying victimization, substance use, and mental health; however, there are few scientifically tested interventions currently available to reduce these disparities. This paper describes our protocol for the study of a Web-accessible game intervention aimed at improving the health of SGMY. Specifically, the outcomes of this pilot study are to assess the feasibility of implementing a game intervention to SGMY and limited efficacy of this intervention: increasing help-seeking–related knowledge, intentions, self-efficacy, and behaviors; increasing productive coping skills use and coping flexibility; and reducing health risk factors and behaviors among SGMY. Our study protocol directly informs the scientific development of a future, larger RCT testing the limited efficacy of our game intervention.

## Abbreviations

GSA: gay-straight alliance or gender and sexuality alliance
LGBTQ: lesbian, gay, bisexual, transgender, and queer
RCT: randomized controlled trial
SGM: sexual and gender minority
SGMY: sexual and gender minority youth
T1: baseline
T2: first follow-up
T3: final follow-up
YRBS: Youth Risk Behavior Survey
